# Continuous Theta-Burst Stimulation in a 9-year-old girl with a history of neurotoxicity after Acute Lymphoblastic Leukemia B

**DOI:** 10.1192/j.eurpsy.2023.359

**Published:** 2023-07-19

**Authors:** A. Moleon, M. Martín-Bejarano, T. Javier, I. Pérez, T. Rosa, M. Garcia-Ferriol, P. Rocío, J. M. Oropesa, N. Javier

**Affiliations:** ^1^ Hospital Universitario Virgen del Rocío; ^2^Instituto Andaluz de Salud Cerebral, Sevilla; ^3^Hospital Universitario 12 de Octubre, Madrid; ^4^Universidad de Cádiz, Cádiz; ^5^Instituto Andaluz de Salud Cerebral, Huelva; ^6^Hospital Universitario Virgen Macarena, Sevilla; ^7^Hospital Juan Ramón Jimenez, Huelva, Spain

## Abstract

**Introduction:**

Transcranial Magnetic Stimulation is a non invasive brain stimulation technique used for several neuropsychiatric conditions. The treatment of Acute Lymphoblastic Leukaemia (ALL) involves many cytotoxic drugs that inhibit the rapid growth of cancer cells, but also damage healthy cells, resulting in a wide range of adverse effects (Śliwa-Tytko et al., 2022). Studies have shown that approximately 10-30% of paediatric ALL patients suffer from psychiatric disorders. Therefore, new therapeutic tools are needed, and repetitive transcranial magnetic stimulation (rTMS) has demonstrated tolerability, effectiveness and safety in children (Allen et al., 2017).

**Objectives:**

We discuss the first case of a 9-year-old girl diagnosed with acute lymphoblastic leukaemia B in who underwent Continuous Theta-Burst Stimulation

**Methods:**

*Case Presentation.* In this study, we describe a case of a 9-year-old girl diagnosed with acute lymphoblastic leukaemia B in November 2016 who completed treatment in July 2019. Since April 2018 she presented symptoms of intracranial hypertension and encephalopathy with behavioural alterations, attention deficit secondary to toxicity. Psychotic outbreaks after toxicity from different treatments was also present. Since starting pericyazine (July 2022) there has been a slight improvement, but her symptoms continue to have a severe impact in her daily functioning. Baseline developmental profile assessed with the Battelle Inventory was significantly below the expected level in all developmental areas except for gross motor skills. *Treatment.* The TMS intervention consisted of the application on right DLPFC (F4), inhibitory cTBS protocol (5Hz bursts and 3 pulses of 50 Hz each). The protocol consisted in delivering 2 sessions per day for 15 days (separated by 55 minutes), 4 minutes per session (3600 pulses/session), 30 sessions in total. An intensity of 100% of resting motor threshold (C4). TMS was performed with the Magventure Magpro X100 MagOption equipment, Cool DB-80 double cone coil. The Child Behaviour Checklist (CBCL) for parents was used to assess intervention effects.

**Results:**

CBCL results reflect improvements in both internalising and externalising total scores after treatment. Specifically, the patient presents clinically significant decreases in several dimensions such as anxious/depressed symptoms, somatic complaints, and social problems. No adverse effects have been reported since the beginning of the intervention.

**Conclusions:**

Internalising and externalising behaviours severity were reduced after 30 TMS sessions. In accordance with the latest systematic reviews on the safety of TMS in the paediatric patient (Zewdie et al, 2020) we propose the development of paediatric guidelines to offer this technique to patients with a history of intolerability or poor drug response.

**Disclosure of Interest:**

None Declared

